# Micronutrient Antioxidants for Men (Menevit^®^) Improve Sperm Function by Reducing Oxidative Stress, Resulting in Improved Assisted Reproductive Technology Outcomes

**DOI:** 10.3390/antiox13060635

**Published:** 2024-05-23

**Authors:** Seiji Ogawa, Kaori Nishizawa, Masumi Shinagawa, Mikiko Katagiri, Hiroyuki Kikuchi, Hideyuki Kobayashi, Hiroaki Yoshida

**Affiliations:** 1 Sendai ART Clinic, 206-13 Nagakecho, Miyagino, Sendai 983-0864, Miyagi, Japan; nishizawa@sendai-art-cl.jp (K.N.); shinagawa@sendai-art-cl.jp (M.S.); micco0904@yahoo.co.jp (M.K.); kikuchi@sendai-art-cl.jp (H.K.); hideyukk@med.toho-u.ac.jp (H.K.); hiroaki@sendai-art-cl.jp (H.Y.); 2 Department of Clinical Regenerative Medicine, Fujita Medical Innovation Center, 1-1-4 Hanedakuko, Ota, Tokyo 144-0041, Japan; 3 Department of Urology, Toho University, 5-21-16 Omori-Nishi, Ota, Tokyo 143-8540, Japan

**Keywords:** folic acid, zinc, antioxidant, semen, reactive oxygen species, male infertility

## Abstract

Oxidative stress (OS) affects men’s health and impairs spermatogenesis. Micronutrient antioxidants are available for male infertility as complemental support; however, their efficacy remains debatable. This study aimed to investigate whether antioxidants can help to reduce sperm OS and improve semen analysis and quality. We included 171 male partners of couples planning to undergo assisted reproductive technology (ART). Male partners, aged 29–41 years, of couples intending to conceive were self-selected to take daily antioxidants (n = 84) containing folic acid and zinc, or not to take antioxidants (n = 52) for 6 months. We analyzed the alterations in serum oxidant levels, sperm parameters, OS, and deoxyribonucleic acid fragmentation after 3 and 6 months. Additionally, implantation, clinical pregnancy, and miscarriage rates after vitrified–warmed embryo transfer were compared between those taking antioxidants and those not taking them after 6 months. In men with high static oxidation–reduction potential (sORP), we observed a significant improvement in sperm concentration and sORP. The high-quality blastocyst rate tended to increase, and implantation and clinical pregnancy rates also significantly increased after 6 months of intervention. The micronutrient antioxidants could improve sperm function by reducing OS and improving ART outcomes. Therefore, micronutrient antioxidants may be a viable treatment option for male infertility.

## 1. Introduction

Infertility is a widespread problem affecting 8–12% of the global population [1]. In approximately 50% of these cases, a male factor, including abnormal semen parameters, such as oligozoospermia, asthenozoospermia, and teratozoospermia; a combination of all three (oligo-astheno-teratozoospermia); or azoospermia, is involved. A male factor alone or in combination is responsible for 30–50% of couples with infertility [2,3], with a severe male factor requiring an intracytoplasmic sperm injection (ICSI) occurring in only up to 30% of couples with male infertility [4,5]. Abnormal spermatogenesis can have multiple origins, such as genetic, environmental, or lifestyle factors [6], with oxidative stress (OS) being the most considerable factor, as implicated in 30% to 80% of all cases [7]. OS, which occurs due to the excessive production of reactive oxygen species (ROS) [8], is a potential marker for the assessment of sperm quality. An association between OS and the poor clinical outcome of in vitro fertilization (IVF) procedures has been observed. ROS in seminal plasma is negatively associated with the fertilization rate, embryo development, and pregnancy rate [9].

All cells in the human body undergo oxidation. Oxidation, which is the loss of electrons from a substance, can lead to the formation of free radicals or ROS, which can damage spermatozoa. Reduction, which is the opposite of oxidation, involves substances gaining electrons. Redox refers to the process of oxidation–reduction. Antioxidative agents can prevent ROS production or deactivate them before they damage cells, deoxyribonucleic acid (DNA), or other cellular components. OS, which results from an imbalance between oxidation (ROS production) and antioxidant levels, is responsible for several detrimental effects, including impaired spermatogenesis, germ and somatic cell apoptosis, oxidative DNA damage, the disruption of gene expression and post-transcriptional gene regulation, and ATP depletion [10,11]. As a result, spermatozoa are functionally impaired, including insufficient phosphorylation of the sperm tail axoneme, lipid peroxidation, and decreased motility and viability [12].

Based on this theory, antioxidants could prevent or treat OS in sperm cells, thereby improving male fertility. Oral supplementation with antioxidants may be used to improve male fertility by reducing OS [13,14]. The updated Cochrane review of antioxidants for male subfertility indicated a small increase in live birth rates with antioxidant use. However, the evidence level is limited because of scarce reports on clinical outcomes [15]. The critical role of OS in male fertility remains a subject of debate among experts. Additionally, excessive antioxidant use may impair fertility through reductive stress [16,17], adding controversy to their use in treating male infertility.

Zinc, which is involved in DNA transcription, is a critical nutrient for spermatogenesis [18]. ROS scavenging has been associated with zinc. Sperm is particularly vulnerable to ROS attack due to its high oxidative phosphorylation activity and low cytoplasmic content, which may lead to reduced sperm quality [19]. Additionally, vitamin E, which is a naturally occurring fat-soluble compound, is a powerful chain-breaking antioxidant that can neutralize free radicals and reduce ROS-induced lipid peroxidation, protecting cell membranes. It also inhibits the lipid peroxidation cascade, enhancing the functions of other antioxidants [20]. In men with infertility, vitamin E inhibits ROS production and protects the components of the sperm plasma membrane against lipid peroxidation [21].

Folic acid acts as a coenzyme in many important one-carbon metabolic reactions that are essential for DNA and ribonucleic acid synthesis, as well as various methylation reactions, and has antioxidant, anticarcinogenic, cardiovascular, and neuroprotective properties [22]. For example, folic acid is an important co-substrate in the remethylation of homocysteine (HCY) to the amino acid methionine [23]. The antioxidant properties of folic acid are mediated by a variety of mechanisms, including a reduction in the plasma concentration of HCY, which can increase the total antioxidant capacity and reduce the formation of ROS [22]. Folic acid, alone or combined with other B vitamins, effectively lowers plasma HCY levels [24,25,26,27,28,29,30,31]. Moreover, antioxidant vitamins, such as vitamins C and E, may have an adjunctive role in preventing HCY-mediated OS [32,33,34,35]. Therefore, due to the relationship between OS and sperm DNA damage that inhibits methylation, scientific evidence indicates that folic acid supplementation as a potent antioxidant protects cells from free radical-induced damage. This supplementation may also improve male fertility by enhancing DNA methylation during spermatogenesis [36], regulating MTHFR expression levels, and reducing testicular apoptotic gene expression in male mice [37].

The 2012 update of the European Association of Urology guidelines for male infertility suggested therapies such as follicle-stimulating hormone, folate, zinc, or anti-estrogens as potentially advantageous for some patients, although scientific evidence to support this empirical approach is limited and the most effective treatment remains unclear [38]. In addition, interventional studies that investigated the efficacy of folic acid and zinc supplementation in the treatment of male infertility are limited. Despite ongoing updates, reports on the efficacy of folic acid and folic acid plus zinc antioxidants in the treatment of male infertility remain controversial and require further clarification. The micronutrient antioxidants for men (Menevit^®^) used in this study contain folic acid, zinc, and the antioxidant vitamins C and E. We hypothesized that micronutrient antioxidants reduce ROS-induced sperm quality damage, thereby improving male fertility and pregnancy outcomes. Therefore, this study aimed to investigate whether antioxidants can help to reduce sperm OS and improve semen analysis and sperm quality. Our findings suggest that in individuals with high sperm oxidative stress, the consumption of micronutrient antioxidants improved serum oxidants and semen parameters, resulting in increased implantation and clinical pregnancy rates compared with those not taking antioxidants.

## 2. Materials and Methods

### 2.1. Study Design and Participants

**Figure 1:**
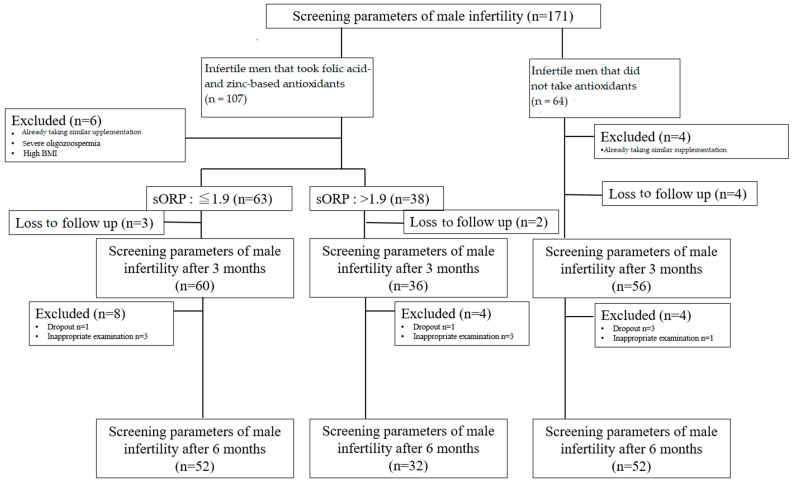
Study flow chart of this longitudinal prospective study.

This single-center, longitudinal, prospective study was performed from 3 October 2020 to 31 March 2022 at the Sendai assisted reproductive technology (ART) clinic in Japan. Figure 1 outlines the study design.

We recruited 171 male partners of couples experiencing infertility after at least 1 year of unprotected intercourse, and thus, requiring IVF-ICSI treatment. Male partners with non-obstructive azoospermia, obstructive azoospermia scheduled for microsurgery, severe oligozoospermia (sperm count less than 1 × 106/mL), an identifiable cause of infertility (leukocytospermia and/or positive sperm culture, epididymal-orchitis, prostatitis, inguinoscrotal surgery, cryptorchidism, varicocele, etc.), higher BMI (>30), endocrine, metabolic, autoimmune, or neoplastic diseases and those already taking antioxidants such as high-dose vitamin-related supplementation were excluded in this study. Female partners with diminished ovarian reserve (serum Anti-Müllerian Hormone concentration < 1 ng/mL) were excluded. As a lifestyle factor, there were none of the heavy smokers defined as smoking ≥30 cigarettes (or 1.5 packs) per day according to self-report. Of the 161 male partners who fulfilled the inclusion criteria, 101 chose to begin the daily micronutrient antioxidants intervention (Menevit^®^, Bayer Yakuhin, Ltd., Tokyo, Japan), whereas 60 declined. Menevit^®^ tablets contain 6 mg lycopene, 30 mg vitamin E, 1.3 mg vitamin B6, 2.4 μg vitamin B12, 180 mg vitamin C, 12 mg zinc, 60 μg selenium, 400 μg folic acid, and 50 mg L-carnitine. Three tablets daily are the recommended dosage. Twenty-five participants did not complete the study, nine were lost to follow-up, and 16 were excluded for failing to comply with the intervention or for having incomplete measurements. Therefore, 136 participants/couples completed the study and were included in the statistical analyses. The oral administration of Menevit^®^ was not associated with any adverse effects. Male partners underwent routine infertility evaluation, including basal follicle-stimulating hormone, luteinizing hormone, testosterone, and basic semen analysis, during the initial visit. Furthermore, serum oxidants, including folate, zinc, vitamin E, and HCY, were analyzed, and zinc, creatinine, spermine, and testosterone, which influence sperm quality, were measured. Male patients underwent examinations of serum oxidants and semen parameters every 3 months from the initial visit.

IVF/ICSI procedures, including ovarian stimulation, oocyte retrieval, and insemination using IVF or ICSI, were performed as previously described [39]. A frozen–thawed embryo transfer (FET), typically utilizing a hormone replacement cycle, was performed as previously described [40]. Briefly, an FET was planned, the endometrium was prepared with a hormone replacement cycle, and transdermal estradiol (0.72 mg, Estrana TAPE, Hisamitsu Pharmaceutical, Tokyo, Japan) was started on CD 3. Progesterone treatment with vaginal progestin tablets (300 mg/day, LUTINUS, Ferring Pharmaceuticals Co., Ltd., Tokyo, Japan; 600 mg/day, Utrogestan, Fuji Pharmaceuticals Co., Ltd., Tokyo, Japan; or 800 mg/day, Luteum, ASKA Pharmaceuticals Co., Ltd., Tokyo, Japan) was started at an endometrial thickness of 8 mm. An FET was scheduled to be performed 5 days after the start of progesterone treatment. We analyzed the IVF/ICSI outcomes regarding the number of oocytes retrieved, number of oocytes in the metaphase II (MII) stage, fertilization rate, and high-quality blastocyst (defined as 4BB or above according to Gardner’s grading scale [41]) rate. The MII rate is the number of oocytes at MII divided by the number of oocytes retrieved. The fertilization rate was calculated as the number of embryos divided by the number of oocytes retrieved for IVF or the number of oocytes retrieved at the MII stage for ICSI. The number of high-quality embryos divided by the number of embryos was used to calculate the high-quality blastocyst rate. We also examined the pregnancy outcomes as a secondary outcome to analyze whether an intervention with micronutrient antioxidants would improve the course of pregnancy after the FET. Therefore, we compared pregnancy outcomes, including implantation, clinical pregnancy, and miscarriage rates, at 6 months after the male patients began taking micronutrient antioxidants from the initial visit between the 84 men with the micronutrient antioxidants and 52 men without the antioxidants. Implantation was defined as the detection of serum human chorionic gonadotropin > 100 mIU/mL, and clinical pregnancy was defined as the detection of an intrauterine sac by transvaginal ultrasound. Miscarriage was defined as the disappearance of a clinical pregnancy, as indicated by the absence of an intrauterine sac on a transvaginal ultrasound.

### 2.2. Semen Sample

Semen samples were collected via masturbation on the first day of the visit, following World Health Organization (WHO) criteria for volume, liquefaction time, pH, white blood cell concentration, spermatozoa concentration, motility progression, morphological (Kruger criteria), and viability [42]. Immediately after completing the semen analysis, an aliquot of semen was collected, and 10 × 10^6^ spermatozoa were used for 8-hydroxy-2′-deoxyguanosine (8-OHdG) and acrosome reaction analysis (in fresh semen), and 2 × 10^6^ spermatozoa were frozen (−70 °C) for the sperm chromosome structure assay.

### 2.3. Measuring Static Oxidation–Reduction Potential (sORP) Using MiOXSYS

Fresh semen samples were used to measure the ORP after liquefaction at room temperature for 30 min. The MiOXSYS^®^ system (Englewood, CO, USA), which is a highly specific in vitro diagnostic tool for measuring the sORP in human semen, was used, as previously described by Agarwal et al. [43,44]. Briefly, 30 μL of liquefied semen sample was applied to the sensor chip and measured with the MiOXSYS^®^ analyzer. The raw ORP value was calculated after 2 min. The raw value was normalized to the total sperm concentration (sORP). In this study, 1.38 mv/106 mL was used as the cut-off value for semen sORP to differentiate between normal and abnormal sperm, and 1.41 mv/106 mL was used as the cut-off value to classify sperm as infertile or fertile based on previous reports and the MiOXSYS system user manual [14,43,45,46]. Higher sperm DNA fragmentation and low fertilization rates were observed in patients with an sORP higher than 1.38 mv/10^6^ mL, which is indicative of male infertility [43,46,47]. However, these cut-off values correlate with sperm parameters but not with the clinical outcomes of ART. Conversely, this interventional study investigated the clinical outcome following IVF-ET and considered different cut-off values. A well-designed study involving Japanese men with infertility reported a cut-off value of 2.59 mv/10^6^ mL, which correlated with semen findings and clinical outcomes. As previously reported [48], a cut-off value of 1.9 mv/10^6^ mL for the sORP correlates with clinical outcomes, resulting in a fertilization rate of 50% or less following ICSI. Therefore, we adopted sORP values of 1.9 mv/10^6^ mL and compared the sperm quality, serum oxidants, 8-OHdG, DNA fragmentation index (DFI), and semen parameters. Furthermore, we analyzed the fertilization, embryo development, embryo quality, and pregnancy outcomes.

### 2.4. Detection of 8-OHdG Levels

The level of 8-OHdG, which is an indicator of oxidative DNA damage, was measured in the seminal plasma to assess OS in a similar method to that previously described [49]. The process involved creating an 8-OHdG–bovine serum albumin complex by dispensing 50 μL of 8-OHdG antigen into a 96-well microplate and incubating it for 1 h at room temperature. Subsequently, the wells were washed four times with a washing solution and blocked with a 1% bovine serum albumin/phosphate-buffered saline solution for 1 h at room temperature. The wells were washed four more times with the washing solution. Subsequently, 50 μL of the specimen or serially diluted standard sample was dispensed into each well, and 50 μL of HRP-labeled anti-8-OHdG antibody (Japan Institute for the Control of Aging, Tokyo, Japan) was continuously dispensed into each well. After mixing, 100 μL of 3,3′,5,5′-tetramethylbenzidine solution was dispensed into each well of the microplate, and the mixture was allowed to react for 1 h at room temperature. The microplate was sealed after washing the wells four times with the washing solution and incubated for 15 min at room temperature in the dark. Finally, 50 μL of 1 M of phosphoric acid was dispensed into each well, and the absorbance was measured using a microplate reader (TECAN, Infinite^®^ 200 PRO, Männedorf, Switzerland) at a measurement wavelength of 450 nm and reference wavelength of 570 nm. The concentration of the sample was calculated from the calibration curve.

### 2.5. Measuring Sperm DNA Fragmentation

DNA fragmentation testing was carried out on a frozen-prepared semen sample by means of the Sperm Chromosome Structure Assay, as described in the WHO Laboratory Manual for the Examination and Processing of Human Semen, 6th ed. (WHO Press: Geneva, Switzerland, 2021; available online: https://www.who.int/publications/i/item/9789240030787; accessed on 27 July 2021). Briefly, 400 µL of an acid solution (0.1% Triton X-100, 0.15 M NaCl, and 0.08 N HCI, pH 1.20, 4 °C) was added to 200 µL of sperm suspension (1 × 10^6^ sperm/mL Tris-NaCl-EDTA (TNE) buffer [0.01 M Tris, 0.15 M NaCl, 1 mM EDTA]) for 30 s, followed by the addition of 1.20 mL acridine orange (AO) dye solution (containing 6 mg purified AO/L (Invitrogen™, Thermo Fisher Scientific), AO buffer (370 mL stock 0.1 M citric acid, 630 mL stock solution of 0.2 M Na_2_HPO_4_, 1 mM disodium EDTA, and 0.15 M NaCl, pH 6.0, 4 °C). Upon excitation at 488 nm, AO bound to double-stranded DNA emits green fluorescence, whereas AO bound to single-stranded DNA emits red fluorescence. The percentage of DNA fragmentation of the total sperm population (percent of the DNA fragmentation index (% DFI)) was determined from cytograms of non-fragmented (green fluorescence) versus fragmented DNA (red fluorescence), quantified by the ratio of red/[red + green] fluorescence. A reference sample (≤15% DFI) was used as a control at the beginning of each experiment to verify the threshold between unfragmented (main cell population) and fragmented sperm.

### 2.6. Ethical Statement

The Ethics Review Committee of Sendai ART Clinic approved this study. After a detailed description of the purpose of this study, written informed consent was obtained from all participants. All experimental procedures were conducted according to the tenets of the Declaration of Helsinki.

### 2.7. Statistical Analysis

Statistical analysis was performed using EZR version 1.51 statistical software [50]. Continuous variables with a normal distribution were presented as the mean ± standard deviation (*p* > 0.05 in the Kolmogorov–Smirnov test or Shapiro–Wilk test [n < 30]). Continuous variables with a non-normal distribution were presented as a median. The continuous variables were compared using Student’s *t*-test or the Mann–Whitney U-test depending on whether they were parametric or non-parametric values, respectively. Comparisons between intervention (≦1.90 and >1.9) and non-intervention or baseline, 3 months, and 6 months were performed using Frieman’s test and the Kruskal–Wallis test for data with a non-normal distribution. Statistical significance was set at *p* < 0.05.

## 3. Results

### 3.1. Baseline Characteristics of the Study Participants

Table 1 shows a comparison of the clinical characteristics between the 136 men with infertility who took antioxidants (n = 84) and those who did not (n = 52). The median age differed significantly between the participants who took antioxidants and those who did not (*p* = 0.0114), whereas the mean BMI showed no significant difference between the two groups (*p* = 0.131). Regarding the serum hormones, significant differences were observed in follicle-stimulating hormone and luteinizing hormone levels between the antioxidant and no-antioxidant groups (*p* = 0.00217 and *p* = 0.0341, respectively), whereas the testosterone levels did not significantly differ (*p* = 0.309). The serum oxidants, including folate, zinc, vitamin E, and total HCY, did not significantly differ between the antioxidant and no-antioxidant groups (*p* = 0.0778, *p* = 0.83, *p* = 0.845, and *p* = 0.957, respectively). Among the semen parameters, significant differences were observed in sperm concentration, total motility, progressive motility, sORP, DFI, zinc, and spermine between the antioxidant and no-antioxidant groups (*p* < 0.001, *p* < 0.001, *p* < 0.001, *p* < 0.001, *p* = 0.014, *p* = 0.0191, and *p* = 0.0337, respectively).

### 3.2. Serum Oxidants and Semen Parameters

The serum oxidants are summarized in Table 2. The serum folate levels did not differ significantly between the men with sORP ≤ 1.9 mv/10^6^ mL that took oral antioxidants, those with sORP > 1.9 mv/10^6^ mL that took antioxidants, and those without antioxidants at baseline. However, the serum folate levels were significantly higher in the men that took the oral antioxidants after 3 and 6 months than in those that did not take the antioxidants. Additionally, a significant increase in the serum folate levels was observed in the men that took the antioxidants after 6 months. The serum zinc levels did not significantly differ between the three groups at baseline and did not increase significantly after 3 or 6 months, regardless of antioxidant use. The serum vitamin E levels did not differ significantly between the men with sORP ≤ 1.9 mv/10^6^ mL that took oral antioxidants, those with sORP > 1.9 mv/10^6^ mL that took antioxidants, and those that did not take antioxidants at baseline. However, the serum vitamin E levels were significantly higher in the men that took oral antioxidants only after 6 months than in those that did not take anti-antioxidants. Additionally, a significant increase in vitamin E levels was observed in the men that took antioxidants, whereas a significant decrease in vitamin E levels was observed in those that did not take antioxidants. The semen parameters are summarized in Table 3. The serum total HCY levels did not significantly differ between the men with sORP ≤ 1.9 mv/10^6^ mL that took oral antioxidants, those with sORP > 1.9 mv/10^6^ mL that took antioxidants, and those that did not take antioxidants at baseline. However, the serum total HCY levels significantly decreased in the men that took oral antioxidants after 3 or 6 months than in those that did not take antioxidants. Additionally, a significant decrease was observed in the total HCY in the men that took antioxidants. The semen volume did not significantly change between the three groups after 3 or 6 months. The semen volume, total motility, and progressive motility did not significantly change between the three groups after 3 or 6 months. In contrast, the men with sORP > 1.9 mv/10^6^ mL that took antioxidants had significantly lower sperm concentrations than those with sORP ≤ 1.9 mv/10^6^ mL that took oral antioxidants and those that did not take antioxidants at baseline. However, the sperm concentration levels in men with sORP > 1.9 mv/10^6^ mL that took antioxidants after 6 months significantly improved more than those at baseline (*p* < 0.05). The sORP in the men with >1.9 mv/10^6^ mL that took antioxidants was significantly higher than the other groups at baseline, and the sORP in the men with sORP >1.9 mv/10^6^ mL that took antioxidants significantly improved after 6 months (*p* < 0.05). However, no significant change was observed in the men with sORP ≤ 1.9 mv/10^6^ mL that took oral antioxidants after 6 months (*p* = 0.441). Furthermore, 8-OHdG did not change between the three groups at baseline, and no significant improvement was observed in the antioxidant and no-antioxidant groups. Additionally, the DFI in the men with sORP > 1.9 mv/10^6^ mL that took antioxidants was significantly higher than the other groups at baseline, and no significant improvement in the DFI was observed in the antioxidant and no-antioxidant groups.

### 3.3. Clinical Outcomes of IVF-ET

The alteration of the ART outcome between taking oral antioxidants and not taking antioxidants are summarized in Table 4. At baseline, no significant differences were observed between the men with sORP ≤ 1.9 mv/10^6^ mL that took oral antioxidants, those with sORP > 1.9 mv/10^6^ mL that took antioxidants, and those with no antioxidants regarding the number of oocytes retrieved, number of MII oocytes, fertilization rate, and high-quality blastocyst rate. Moreover, no changes were observed in those with or without oral antioxidants after 6 months. Furthermore, the implantation and clinical pregnancy rates did not significantly differ between the three groups before the intervention, although the intervention with oral antioxidants for 6 months significantly improved the implantation and clinical pregnancy rates in the men with sORP > 1.9 mv/10^6^ mL that took antioxidants (*p* < 0.05 and *p* < 0.05, respectively). The men with sORP > 1.9 mv/10^6^ mL that took antioxidants had the highest improved implantation and clinical pregnancy rates compared with the other groups (*p* < 0.05 and *p* < 0.05, respectively). No significant differences were observed in miscarriage rates between the three groups at baseline, and no change was observed in those that took or did not take oral antioxidants after 6 months.

## 4. Discussion

The present interventional study supported the hypothesis that antioxidants are associated with a trend toward improving reproductive outcomes in men with infertility undergoing IVF-ICSI. Concretely, our findings suggest that in individuals with high sperm OS (high sORP), the consumption of micronutrient antioxidants improved serum oxidants and semen parameters, resulting in increased implantation and clinical pregnancy rates compared with those not taking antioxidants.

Micronutrient deficiencies, such as folic acid and zinc, may be involved in male infertility, which is a complex problem that afflicts many couples all over the world. Investigations examined the effects of folic acid and folic acid plus zinc antioxidants in reducing ROS to improve sperm characteristics and pregnancy outcomes in men with infertility. OS, mainly as a result of elevated ROS levels, can compromise sperm viability and decrease the sperm count/concentration [51]. Our study results support previous findings that micronutrient antioxidants, including folic acid and zinc, have a beneficial effect on sperm concentration but not on sperm count. Ebisch et al. described a significant increase in sperm concentration in infertile men taking folic acid- and zinc-based supplements [52]. Raigani et al. also showed that folic acid- and zinc-based supplements improve sperm concentration in infertile men taking only folic acid supplementation [53]. However, a recent meta-analysis of five randomized controlled trials (RCTs) demonstrated that folic acid- and zinc-based antioxidants do not have a significant effect on sperm concentration in men with infertility (MD, 0.95; 95% confidence interval [CI], −4.54 to 6.45; *p* = 0.73) [54]. This contradictory result can be attributed to the 6-month duration of treatment with folic acid- and zinc-based antioxidants in our study, which may have influenced the improvements compared with the shorter treatment durations examined in previous studies. This finding means that long-term intervention, such as 6 months with folic acid- and zinc-based antioxidants, may have improved the turnover of spermatogenesis because one piece of evidence reported no relation between folic acid- and zinc-based antioxidants and sperm features, especially for durations shorter than one cycle of spermatogenesis [55]. Moreover, the micronutrient antioxidants used contained folic acid and coenzymes in the HCY metabolism pathway, efficiently lowering HCY levels, may have an adjunctive role in preventing HCY-mediated OS and can also improve male fertility by enhancing DNA methylation during spermatogenesis. OS can also affect sperm production in the testis, resulting in decreased sperm count and oligozoospermia [56].

These findings are important, as immature and morphologically abnormal sperm are considered the main endogenous sources of ROS, potentially leading to reduced sperm genomic integrity [57]. OS is related to sperm DNA fragmentation, gene mutations, and genetic disorders [12]. Previously, some researchers reported that OS is associated with infertility problems in males, such as reduced sperm motility, sperm DNA damage, and an increased risk of recurrent miscarriages [12,58,59], since excessive OS can have detrimental effects on fertility, pregnancy outcomes, and the genetic health of newborns [60]. Furthermore, an equilibrium between elevated ROS and diminished antioxidant defenses induces oxidative stress, which negatively alters sperm motility and DNA integrity [61]. ORP is a direct measure of OS or redox imbalance in biological samples. As an indicator of OS, ORP has been associated with the severity of pathogenesis owing to the reflection of cellular damage [62]. Male patients with infertility have higher sORP levels than fertile individuals [46,63]. Additionally, sORP negatively correlates with total sperm count, motility, and morphology [7,46,63,64], but positively correlates with sperm DNA fragmentation [63,64]. Thus, OS in semen may be the cause of infertility associated with reduced basic semen parameters and the genomic integrity of spermatozoa. In this study, when the sORP was >1.9 mv/10^6^ mL, the oral intake of micronutrient antioxidants significantly gradually reduced the OS in semen over 3 and 6 months. Differences were observed between the two oxidative markers, sORP and 8-OHdG. The participants were divided into two groups using 1.9 mv/10^6^ mL as the cut-off value based on their pre-interventional ORP levels, and the pre- and post-interventional 8-OhdG levels did not differ between the groups. Additionally, no apparent correlations were observed between the ORP and 8-OhdG in men that underwent pre- and post-intervention. Hence, these two markers might not represent the same aspect of OS in sperm. ORP, also known as redox balance, is a direct biomarker of OS, as it describes the relative proportion of ROS and antioxidative substances [65]. In contrast, 8-OHdG is generally recognized as a key biomarker of oxidative damage [66,67], and sperm 8-OHdG levels have been associated with semen parameters in patients with infertility [68,69]. In this study, these two markers did not yield consistent results, consistent with the report of other studies suggesting they cannot be parallelly used as a single assay to diagnose OS [70,71]. Furthermore, the DFI is a biomarker used to detect sperm DNA fragmentation or damage and is an advanced test of sperm function correlated to fertility outcomes [72,73]. Previous reports have shown that sperm DFI negatively relates to sperm concentration [74,75] and positively correlates with sORP [76]. Sperm DFI is independent of semen parameters and is a better predictor of male fertility than routine semen parameters [77]. In a review of 20 studies, Gharagozloo et al. reported that oral antioxidant supplementation was associated with a significant reduction in OS, improvement in sperm function parameters, and decreased sperm DNA fragmentation [78]. In particular, folic acid- and zinc-based antioxidants have a positive impact on sperm DNA fragmentation [78]. On the other hand, A recent RCT reported a negative impact; taking folic acid- and zinc-based antioxidants led to a significant increase in DNA fragmentation compared with taking a placebo (29.7% versus 27.2%; mean difference 2.4% [95% CI, 0.5–4.4%]) [79]. In this study, pre-interventional DFI levels were significantly higher in male patients with sORP >1.9 mv/10^6^ mL, but the oral intake of folic acid- and zinc-based antioxidants did not significantly reduce the DFI. The effect of folic acid- and zinc-based antioxidants on DNA fragmentation remains controversial, and even considering the turnover in spermatogenesis, oral intake for 6 months reduced OS but did not improve the DNA fragmentation. Additionally, even in the group (>1.9 mv/10^6^ mL of sORP) with the highest DFI in this study, the values did not exceed the standard criteria of the test, and perhaps because the group originally had relatively low DFI levels, the effect of micronutrient antioxidants on the DFI may be unclear.

A recent Cochrane review that comprised 48 RCTs examined the effect of antioxidants on male infertility elucidated a significant improvement in clinical pregnancy rates (odds ratio [OR] 3.43, 95% CI 1.92–6.11, *p* < 0.001) and live birth rates (OR 4.21, 95% CI 2.08–8.51, *p* < 0.001) [80]. Some studies also elucidated the beneficial efficiency of folic acid and zinc antioxidants on pregnancy outcomes in infertile men. Well-designed RCTs, such as the Folic Acid and Zinc Supplementation RCT (FAZST) in couples that underwent infertility therapy, reported no significant improvements in semen parameters or pregnancy outcomes for male partners that took high-dose folic acid- (5 mg) and zinc-based (30 mg) antioxidants for 6 months than for those that took a placebo [79,81]. However, a further prospective cohort study found that 3 months of folic acid- (400 mg) and zinc-based (15 mg) antioxidants in men with infertility that underwent IVF-ICSI resulted in improved semen parameters or pregnancy outcomes [82]. In this study, pregnancy outcomes after ET before intervention did not significantly differ between the three groups. However, in the men with sORP > 1.9 mv/10^6^ mL, the oral intake of micronutrient antioxidants containing folic acid and zinc for 6 months significantly improved the implantation and clinical pregnancy rates compared with the other groups. We intervened with folic acid- and zinc-based antioxidants of approximately the same amount of folic acid (400 mg) and zinc (12 mg) as those used in the study by Scaruffi et al. [82]. Conversely, the high-dose intake of zinc lowers sperm quality [54]; therefore, the results of Scaruffi et al. [82] and our study might differ from those of the FAZST [79]. The folic acid- and zinc-based antioxidants in the FAZST did not exhibit a positive impact on sperm parameters or pregnancy outcomes [79]; however, we speculated that differences in the study design may have been the reason for the difference as the intervention was not used for a population with poor semen quality, such as >1.9 mv/10^6^ mL of sORP. Moreover, folate, zinc, and a combination of seven types of micronutrients may have contributed to a synergistic effect, including the reduction in HCY. This study is promising; however, further research is needed to clarify the appropriate dosage and duration of micronutrient antioxidants, as well as the underlying mechanisms. Health professionals should consider recommending folic acid- and zinc-based antioxidants to men with infertility, especially those with high sORP, undergoing IVF-ICSI while addressing other potential contributing factors to infertility.

Oxidative stress induces epigenetic changes in germ cells, altering DNA methylation and histone modifications [12]. This can impact gene expression, potentially influencing the health of the next generation, as well as self-fertility. Epigenetic changes in germ cells, specifically alterations in DNA methylation patterns and histone modifications, can occur due to these influencing the OS [83,84]. In this study, although either the sperm DFI or 8OHdG levels, which are markers of sperm oxidative DNA damage, were not changed, intervention with micronutrient antioxidants improved only the sORP. Therefore, it is possible that micronutrient antioxidants directly improved the outcome of the IVF/ICSI cycle, although it is also possible that DNA methylation abnormalities caused by OS were rescued by micronutrient antioxidants and upregulated the high-quality blastocyst rate and implantation rate.

This study had some limitations. First, this study was a patient-oriented intervention trial, which is problematic because of the lack of an aligned patient background and a placebo, which may introduce many confounding factors. Second, sORP was used as a parameter of sperm quality; however, most participants in our study met WHO criteria, potentially leading to low sORP levels. Third, the total intake of folic acid and zinc could not be calculated because of a lack of dietary intake data. Fourth, our study focused on folic acid and zinc antioxidants; however, other antioxidants, such as vitamins B and C, may have influenced the results. Fifth, OS influences epigenetic alterations by impacting histone modifications, such as acetylation and methylation, thereby shaping the gene expression patterns essential for meiotic progression. The intricate interplay between meiotic phases and epigenetic alterations significantly influences male fertility, and the altered epigenetic signature as the malefactor may disturb the embryo following fertilization [12,83,84,85]. However, since this study did not analyze epigenetic changes in sperm, it is unclarified whether micronutrient antioxidants had an epigenetic effect on the improved clinical outcome. Sixth, when considering the impacts of folic acid- and zinc-based antioxidants, the MTHFR gene polymorphism must be considered because some researchers have reported that two polymorphisms in the MTHFR gene (677CT and 677TT) affect male fertility [86,87,88]; however, our analysis did not investigate the genetic background. Further large-scale, prospective, multicenter studies are needed to solidify our findings because this study was not a real-world analysis, but only an analysis in a limited population.

Nevertheless, this study had several strengths. It is the strongest point that two well-designed studies were undertaken using Menevit^®^ as micronutrient antioxidants. One case–control study described that Menevit^®^ resulted in significant improvements in sperm DNA integrity and a reduction in seminal ROS production and apoptosis [89]. Another prospective randomized double-blind placebo-controlled trial described that Menevit^®^ had a statistically significant improvement in pregnancy rate following the IVF/ICSI program compared with the control group [90]. However, these two studies investigated the outcome of sperm quality by Menevit and the clinical outcomes of IVF/ICSI treatment by Menevit, respectively, and this was the first study to analyze two outcomes, providing more support for the previous two reports. Furthermore, this study analyzed the monitoring for various markers after the oral administration of micronutrient antioxidants to confirm good compliance, and no other study has confirmed the effects of the intervention in detail. Therefore, the results of this study are valuable because of the veracity of what happened in vivo.

In conclusion, this prospective interventional study revealed significant improvements in sperm characteristics and pregnancy outcomes following IVF/ICSI-ET when micronutrient antioxidants were used. The speculated mechanisms are as follows: (1) oral intake of micronutrient antioxidants systemically increases antioxidants; (2) these antioxidants focally reduce semen OS, but do not prevent DNA damage; and (3) the high-quality blastocyst rate tends to increase after the intervention, and the pregnancy rate improves. These findings may support the use of micronutrient antioxidants in male partners who undergo infertility treatment, although medical professionals should consider other factors contributing to infertility.

## Figures and Tables

**Table 1:** Patient characteristics.

	Oral Antioxidants (n = 84)	No Antioxidants (n = 52)	*p*-Value
Age (median, range)	37 (31–41)	33 (29–37)	0.0114 ^a^
Body mass index (mean ± SD)	23.5 ± 3.2	24.4 ± 2.9	0.131 ^b^
Serum hormone			
FSH (mIU/mL, mean ± SD)	4.3 (3.2–5.9)	3.4 (2.4–4.6)	0.00217 ^a^
LH (mIU/mL, mean ± SD)	2.9 (2.3–4.1)	2.6 (2.1–3.1)	0.0341 ^a^
Testosterone (ng/mL, mean ± SD)	5.1 (4.2–6.7)	4.9 (3.8–6.3)	0.309 ^a^
Serum oxidants			
Folate (ng/mL)	6.9 (5.1–9.0)	5.8 (5.0–7.7)	0.0778 ^a^
Zinc (μg/dL)	80.0 (73.5–92.0)	84.5 (69.8–91.5)	0.83 ^a^
Vitamin E (mg/dL)	1.10 (0.94–1.30)	1.19 (1.00–1.43)	0.0845 ^a^
Total homocysteine (nmol/mL)	12.4 (10.8–14.8)	12.0 (10.8–15.0)	0.957 ^a^
Semen parameters			
Semen volume (mL)	3.0 (2.0–4.0)	2.5 (2.0–3.7)	0.239 ^a^
Sperm concentration (10^6^ per mL)	28.7 (10.8–66.0)	90.0 (64.7–127.6)	<0.001 ^a^
Total motility (%)	55.0 (34.0–75.5)	81.5 (71.0–92.3)	<0.001 ^a^
Progressive motility (%)	44.0 (25.0–61.0)	64.0 (53.5–77.3)	<0.001 ^a^
Normal forms (%)	3.0 (2.0–5.0)	4.0 (3.0–5.0)	0.0523 ^a^
sORP (mV/10^6^)	0.81 (0.23–2.77)	0.27 (0.12–0.45)	<0.001 ^a^
OHdG (ng/mL)	13.4 (10.5–18.1)	11.9 (9.6–15.6)	0.072 ^a^
DFI (%)	13.8 (9.0–21.6)	10.1 (7.3–13.8)	0.014 ^a^
Zinc (mg/dL)	11.4 (6.5–16.8)	15.4 (9.2–21.1)	0.0191 ^a^
Creatinine (μg/mL)	162.2 (122.1–209.3)	158.7 (135.5–210.1)	0.677 ^a^
Spermine (mM)	0.70 (0.56–0.85)	0.54 (0.34–0.83)	0.0337 ^a^
Testosterone (ng/mL)	23.5 (15.8–30.9)	21.4 (14.9–28.6)	0.478 ^a^

^a^: Mann–Whitney U-test, ^b^: Student’s *t*-test.

**Table 2:** The alteration of serum oxidant between those that took oral antioxidants and those that did not take antioxidants.

		Oral Antioxidants (n = 84)		No Antioxidants (n = 52)	*p*-Value
		sORP: ≤1.9 mv/10^6^ mL (n = 52)	sORP: >1.9 mv/10^6^ mL (n = 32)		
Serum Oxidants					
Folate	Baseline	6.8 (5.3–9.0)	7.0 (5.1–8.5)	5.8 (5.0–7.7)	0.183 **
	After 3 months	16.3 (13.0–19.7)	17.4 (15.4–20.0)	6.3 (5.1–8.2)	<0.001 **
	After 6 months	15.5 (11.5–20.0)	16.9 (14.0–19.0)	5.6 (4.5–7.2)	<0.001 **
*p*-value		<0.001 *	<0.001 *	0.073 *	
Zinc	Baseline	85.0 (76.8–92.3)	76.0 (71.5–91.3)	84.5 (69.8–91.5)	0.344 **
	After 3 months	85.0 (79.8–97.3)	83.0 (73.8–94.3)	79.0 (69.5–90.8)	0.257 **
	After 6 months	87.5 (77.3–101.0)	80.5 (73.0–92.8)	82.0 (70.3–92.5)	0.191 **
*p*-value		0.136 *	0.050 *	0.432 *	
Vitamin E	Baseline	1.10 (0.95–1.30)	1.10 (0.90–1.32)	1.19 (1.00–1.43)	0.249 **
	After 3 months	1.24 (1.10–1.40)	1.125 (1.06–1.42)	1.11 (0.97–1.32)	0.107**
	After 6 months	1.24 (1.05–1.37)	1.28 (1.06–1.44)	1.10 (0.97–1.23)	<0.05 **
*p*-value		<0.001 *	<0.001 *	<0.001 *	
Total homocysteine	Baseline	13.0 (11.2–14.8)	12.3 (10.7–14.2)	12.0 (10.8–15.0)	0.843 **
	After 3 months	10.7 (9.75–12.0)	10.5 (9.5–11.7)	12.5 (10.8–15.7)	<0.001 **
	After 6 months	11.0 (9.68–12.1)	11.3 (10.2–12.2)	13.5 (11.0–16.1)	<0.001 **
*p*-value		<0.001 *	<0.001 *	0.518 *	

* Frieman’s test, ** Kruskal–Wallis test.

**Table 3:** The alteration of semen parameters between those that took oral antioxidants and those that did not take antioxidants.

		Oral Antioxidants (n = 84)		No Antioxidants (n = 52)	*p*-Value
		sORP: ≤1.9 mv/10^6^ mL (n = 52)	sORP: >1.9 mv/10^6^ mL (n = 32)		
Semen Parameters					
Semen volume (mL)	Baseline	2.7 (2.0–3.4)	3.6 (2.6–5.0)	2.5 (2.0–3.7)	<0.05 **
	After 3 months	2.6 (2.0–3.5)	3.3 (2.8–4.6)	2.4 (1.8–3.5)	<0.05 **
	After 6 months	2.6 (2.0–3.2)	3.6 (2.3–4.4)	3.0 (2.0–3.7)	0.096 **
*p*-value		0.691 *	0.227 *	0.889 *	
Sperm concentration (10^6^ per mL)	Baseline	53.3 (28.5–91.3)	8.4 (4.1–17.6)	90.0 (64.7–127.6)	<0.001 **
	After 3 months	48.3 (26.6–81.2)	10.0 (6.6–18.7)	89.8 (55.2–130.2)	<0.001 **
	After 6 months	49.3 (28.6–68.9)	14.3 (5.3–25.9)	76.7 (50.9–130.2)	<0.001 **
*p*-value		0.186 *	<0.05 *	0.272	
Total motility (%)	Baseline	64.5 (42.5–78.3)	50.5 (25.0–67.3)	81.5 (71.0–92.3)	<0.001 **
	After 3 months	63.5 (41.8–77.3)	45.0 (30.3–61.0)	83.0 (66.8–93.3)	<0.001 **
	After 6 months	58.0 (38.8–71.8)	45.5 (25.5–66.3)	80.5 (67.8- 91.0)	<0.001 **
*p*-value		0.858	0.306	0.520	
Progressive motility (%)	Baseline	50.0 (27.8–62.8)	40.0 (17.3–52.0)	64.0 (53.5–77.3)	<0.001 **
	After 3 months	51.0 (29.5–64.3)	37.0 (19.8–52.3)	62.5 (51.0–75.5)	<0.001 **
	After 6 months	44.5 (29.8–58.3)	36.5 (20.8–58.5)	62.5 (51.8–75.7)	<0.001 **
*p*-value		0.966 *	0.798 *	0.227	
sORP	Baseline	0.33 (0.15–0.70)	3.66 (2.00–7.06)	0.27 (0.12–0.45)	<0.001 **
	After 3 months	0.34 (0.12–0.65)	2.78 (1.58–5.00)	0.24 (0.10–0.61)	<0.001 **
	After 6 months	0.40 (0.17–0.66)	1.53 (1.06–5.19)	0.36 (0.18–0.57)	<0.001 **
*p*-value		0.441 *	<0.05 *	0.095 *	
8-OHdG	Baseline	14.4 (10.6–18.1)	13.2 (9.3–17.9)	11.9 (9.6–15.6)	0.196 **
	After 3 months	14.1 (10.9–16.7)	14.3 (11.4–16.5)	14.1 (9.9–17.9)	0.926 **
	After 6 months	14.6 (11.2–17.7)	13.5 (10.2–16.0)	11.9 (8.6–16.2)	0.084 **
*p*-value		0.841 *	0.519 *	0.174 *	
DFI	Baseline	10.9 (7.9–17.2)	15.2 (12.1–23.8)	10.1 (7.3–13.8)	<0.001 **
	After 3 months	12.6 (7.3–18.2)	15.3 (10.4–22.7)	9.5 (6.5–14.2)	<0.001 **
	After 6 months	13.0 (7.7–21.5)	15.3 (10.8–23.9)	10.2 (6.5–18.6)	<0.001 **
*p*-value		0.116 *	0.261 *	0.334 *	
Zinc	Baseline	10.5 (6.3–16.5)	13.4 (9.8–18.8)	15.4 (9.2–21.1)	<0.05 **
	After 3 months	11.7 (7.1–16.7)	14.5 (11.1–17.1)	14.8 (9.7–19.5)	0.0583 **
	After 6 months	11.2 (7.2–14.1)	12.4 (9.5–16.8)	15.1 (11.1–18.8)	<0.05 **
*p*-value		0.584 *	0.911 *	0.779 *	
Creatinine	Baseline	176.7 (135.4–208.6)	150.7 (112.0–219.0)	158.7 (135.5–210.1)	0.390 **
	After 3 months	162.1 (116.9–196.0)	130.6 (111.7–202.4)	154.8 (128.4–211.1)	0.650 **
	After 6 months	159.7 (126.2–191.3)	138.1 (98.2–201.4)	159.8 (126.9–193.1)	0.432 **
*p*-value		0.050 *	0.911 *	0.050 *	
Spermine	Baseline	0.725 (0.630–0.853)	0.635 (0.488–0.863)	0.540 (0.335–0.832)	0.066 **
	After 3 months	0.695 (0.355–0.862)	0.630 (0.408–0.820)	0.880 (0.388–0.873)	0.881 **
	After 6 months	0.695 (0.478–0.873)	0.625 (0.345–0.885)	0.615 (0.430–0.875)	0.733 **
*p*-value		0.827 *	0.902 *	0.061 *	
Testosterone	Baseline	23.6 (15.8–31.9)	22.6 (16.1–30.6)	21.4 (14.9–28.6)	0.676 **
	After 3 months	25.8 (15.8–37.3)	20.7 (16.2–27.1)	19.9 (14.1–35.8)	0.728 **
	After 6 months	23.1 (15.8–32.4)	21.5 (14.8–28.4)	21.7 (15.5–30.7)	0.650 **
*p*-value		0.694 *	0.102 *	1.000 *	

* Frieman’s test, ** Kruskal–Wallis test.

**Table 4:** The alteration of ART outcome between those that took oral antioxidants and those that did not take antioxidants.

		Oral Antioxidants (n = 84)		No Antioxidants (n = 30)	*p*-Value
		sORP: ≤1.9 mv/10^6^ mL (n = 52)	sORP: >1.9 mv/10^6^ mL (n = 32)		
Number of total oocytes retrieved	Baseline	6.0 (4.0–10.0)	6.0 (2.0–10.0)	7.0 (3.5–13.5)	0.728 **
	After 6 months	4.0 (2.0–6.0)	2.5 (1–9.8)	5.0 (4.0–7.0)	0.296 **
*p*-value		<0.001 *	0.166 *	0.606 *	
Number of MII oocytes	Baseline	5.0 (3.0–8.0)	5.0 (2.0–9.0)	6.0 (3.5–10.0)	0.733 **
	After 6 months	3.0 (2.0–5.0)	2.0 (1.0–7.5)	4.0 (3.0–6.0)	0.324 **
*p*-value		<0.001 *	0.154 *	0.538 *	
Fertilization rate (%)	Baseline	80.0 (60.0–100.0)	75.0 (50.0–92.5)	84.6 (66.7–88.2)	0.496 **
	After 6 months	66.7 (50.0–100.0)	70.7 (50.0–100.0)	77.5 (75.0–83.3)	0.592 **
*p*-value		0.0988 *	0.808 *	0.979 *	
High-quality blastocyst rate (%)	Baseline	20.0 (0–50.0)	33.3 (0–55.3)	43.8 (0–62.8)	0.425 **
	After 6 months	20.0 (0–60.0)	50.0 (26.8–50.0)	40.0 (8.4–79.9)	0.695 **
*p*-value		0.726 *	0.709 *	0.712 *	
Implantaion rate (%)	Baseline	38.9%	47.1%	35.9%	0.529 ***
	After 6 months	53.8%	76.2%	33.3%	<0.05 ***
*p*-value		0.132 ***	<0.05 ***	1.00 ***	
Clinical pregnancy rate (%)	Baseline	25.9%	38.2%	28.2%	0.395 ***
	After 6 months	35.9%	71.4%	22.2%	<0.05 ***
*p*-value		0.301 ***	<0.05 ***	0.753 ***	
Miscarraige rate (%)	Baseline	57.1%	38.5%	18.2%	0.0724 ***
	After 6 months	21.4%	26.7%	50%	0.572 ***
*p*-value		0.05 ***	0.689 ***	0.516 ***	

* Frieman’s test, ** Kruskal–Wallis test, *** Fisher exact test.

## Data Availability

The datasets generated and/or analyzed during this current study are not publicly available due to participant privacy, but they are available from the corresponding author on reasonable request.
