# Correction: Ogawa et al. Micronutrient Antioxidants for Men (Menevit^®^) Improve Sperm Function by Reducing Oxidative Stress, Resulting in Improved Assisted Reproductive Technology Outcomes. *Antioxidants* 2024, *13*, 635

**DOI:** 10.3390/antiox13121569

**Published:** 2024-12-20

**Authors:** Seiji Ogawa, Kaori Nishizawa, Masumi Shinagawa, Mikiko Katagiri, Hiroyuki Kikuchi, Hideyuki Kobayashi, Hiroaki Yoshida

**Affiliations:** 1 Sendai ART Clinic, 206-13 Nagakecho, Miyagino, Sendai 983-0864, Miyagi, Japan; nishizawa@sendai-art-cl.jp (K.N.); shinagawa@sendai-art-cl.jp (M.S.); micco0904@yahoo.co.jp (M.K.); kikuchi@sendai-art-cl.jp (H.K.); hideyukk@med.toho-u.ac.jp (H.K.); hiroaki@sendai-art-cl.jp (H.Y.); 2 Department of Clinical Regenerative Medicine, Fujita Medical Innovation Center, 1-1-4 Hanedakuko, Ota, Tokyo 144-0041, Japan; 3 Department of Urology, Toho University, 5-21-16 Omori-Nishi, Ota, Tokyo 143-8540, Japan

In the originally published article [1], an error occurred regarding authorship and ethical approvals. After publication, the authors Kuniaki Ota and Toshifumi Takahashi, affiliated with Fukushima Medical University, were included as co-authors due to a misunderstanding. The study was conducted as a prospective study, for which ethical approval from Fukushima Medical University was not applicable (as a retrospective study), and their contributions did not meet the criteria for authorship.

To address this, the authorship, Author Contributions, and ethical approval statements have been corrected to remove Kuniaki Ota and Toshifumi Takahashi and their affiliations, and reference only the ethical approval from Sendai ART Clinic.

The corrected Author Contributions statement and Institutional Review Board Statement appear below.

**Author Contributions**: Conceptualization, S.O.; methodology, K.N.; software, H.K. (Hiroyuki Kikuchi); validation, M.S. and M.K.; formal analysis, S.O.; investigation, H.K. (Hideyuki Kobayashi); resources, H.K. (Hideyuki Kobayashi); data curation, S.O.; writing—original draft preparation, S.O.; writing—review and editing, S.O.; visualization, H.K. (Hideyuki Kobayashi); supervision, H.Y.; project administration. All authors have read and agreed to the published version of the manuscript.

**Institutional Review Board Statement**: This study was conducted in accordance with the Declaration of Helsinki and approved by the Institutional Review Board of Sendai ART Clinic (approval number: 202001).

**Text Correction**

“The Ethics Review Committee of Sendai ART Clinic and Fukushima Medical University, Fukushima, Japan, approved this study.” was changed to “The Ethics Review Committee of Sendai ART Clinic approved this study.” in Section 2.6.

The authors state that the scientific conclusions are unaffected. This correction was approved by the Academic Editor. The original publication has also been updated.
